# The combination of olive oil and Lepidium sativum improves the deleterious effects resulting from dexamethasone-induced osteoporosis in rats

**DOI:** 10.1186/s40001-022-00904-8

**Published:** 2022-11-28

**Authors:** Dalia M. Badary, Heba A. Galal, Mahmoud H. Abdelraheim, Mohamed I. Sedeek, Nesma M. Mohamed, Zakaria Y. Abd Elmageed, Magda M. Y. Farrag

**Affiliations:** 1 Pathology Department, Faculty of Medicine, Assiut University, Asyût, 71526 Egypt; 2 Pharmacology Department, Faculty of Veterinary Medicine, Assiut University, Asyût, 71526 Egypt; 3 Pharmacology Department, Faculty of Medicine, Assiut University, Asyût, 71526 Egypt; 4 Clinical Pathology Department, Faculty of Medicine, Assiut University, Asyût, 71526 Egypt; 5 Pharmacognosy Department, Faculty of Pharmacy, Assiut University, Asyût, 71526 Egypt; 6 Department of Pharmacology, Edward Via College of Osteopathic Medicine, University of Louisiana at Monroe, Monroe, LA 71203 USA; 7 Pharmacology Department, Faculty of Medicine, Assiut University, Asyût, 71526 Egypt

**Keywords:** Osteoporosis, rats, olive oil, Lepidium sativum, Alendronate, Osteopontin

## Abstract

**Introduction:**

Osteoporosis is characterized by deterioration of bone microarchitecture and reduced bone mass and can increase the risk of fracture. To reduce this risk, the aim of this study was to compare the combination effects of olive oil and *Lepidium sativum* compared to the conventional drug therapy alendronate.

**Methods:**

Osteoporosed-induced rat model was established by administration of dexamethasone in female adult albino rats. The serum level of Ca^2+^, P^3+^, and osteocalcin was assessed. In addition, histopathological changes and immunohistochemical expression of osteopontin within bone specimens were performed.

**Results:**

Our results showed that a combination of olive oil and Lepidium sativum had a beneficial therapeutic effect in the treatment of osteoporosis as compared to alendronate therapy. This was demonstrated by increase of serum Ca^2+^, P^3+^, and osteocalcin levels in treated compared to control groups. Intriguingly, the highest effect was noticed in rats that received a combination of olive oil and Lepidium sativum compared to the individual treatment. This was reflected by an increase in the cortical bone thickness and a decrease in immunohistochemical expression of osteopontin compared to individual treated groups.

**Conclusion:**

We concluded that the administration of a combination of olive oil and Lepidium sativum improves bone mineral health and intensity and reduces the risk of osteoporosis in a rat model.

## Introduction

Osteoporosis is a multifactorial skeletal disorder, characterized by deterioration of bone microarchitecture and reduced bone mass. This predisposes the bones to increase the risk of bone fracture. Because it develops with no symptoms and stays unnoticed for quite a while, it is called a “*silent disease.”* Soon after, the patient usually presents with one or more bone fractures [1, 2]. It was reported that one in each three women and one of every five men over 50-year-old will suffer a fracture due to osteoporosis. This ratio increases to one each two ladies and one out of three men at age of 60-year-old or more. Approximately, 1.6 million hip fractures are reported every year and the incidence rate will increase 4 times by 2050 [3, 4]. The World Health Organization (WHO) operationally defines osteoporosis as a decrease in bone density, where the standard deviation of this density was 2.5 less than that of the mean for healthy adults who has the same sex, which also referred to be a *T-score of − 2.5*. Postmenopausal ladies with low bone thickness are at higher risk of osteoporosis. Over half of fractures among postmenopausal ladies, including hip fractures, had low bone density [5, 6].

As a result, growing efforts have been taken to treat and reduce the risk of osteoporosis. Toward this goal, a number of natural and semisynthetic products are developed to improve the bone structures and functions. One of these products is oleuropein, which is the main phenolic compound of virgin olive oil. This active constituent improves the bone health by raising the formation of osteoblasts suggesting that oleuropein intake may have preventive role against the bone loss associated with aging and osteoporosis [7]. Not only olive oil enhances the intestinal absorption of calcium [7] but also is as a source of gamma-linolenic acid (GLA), which decreases the excretion of calcium and inhibits bone resorption [8, 9]. Phenolic alcohols, such as tyrosol and hydroxytyrosol, are one of the most important groups of bioactive secondary metabolites in virgin olive oil [10, 11]. Administration of both alcohols in the diet prevents the inflammatory-induced bone mass loss in ovariectomized rats and increases their osteoblast activity by elevating plasma level of osteocalcin [12, 13]. Flavonoids, which are abundant in olive oil, decrease the differentiation of both bone marrow mononuclear cells into osteoclasts [14]. *Lepidium sativum* leaves consist of bioactive and important minerals which positively affect bone density due to their high content of calcium [15]. In addition, it increases serum and liver alpha-linolenic acid (ALA), docosahexaenoic acid (DHA), and eicosapentaenoic acid (EPA), together they improve bone health [16, 17]. Therefore, the present study was conducted to compare the therapeutic effect of using olive oil and Lepidium sativum individually and in combination compared to alendronate as a conventional prescribed drug for treatment of osteoporosis.

## Materials and methods

### Animals and induction of osteoporosis

All experiments were performed incompliance with the relevant laws, faculty guidelines, and the Research Ethical Committee. Faculty of Medicine, Assiut University, approved the experiments, IRBno: IRB17101923. Forty-eight female adult albino rats weighing 200–250 g were obtained from the animal house of the Faculty of Medicine, Assiut University. The rats were 2.5–3 months of age before the induction of osteoporosis by dexamethasone. Rats were housed in the animal house at room temperature and maintained at 22–24 °C. Animals were fed ad libitum on a commercial pellet ration and kept under normal 12 h light/dark cycle. Osteoporosis was induced by the administration of dexamethasone sodium phosphate 7 mg/kg b.w. (Amriya Pharma, Cairo, Egypt), injected intramuscularly once a week for 3 consecutive weeks [18]. Urethane was the anesthetic agent used by an IP injection, in a dose of 1.4 g/kg.

### Experimental design

Animals were divided into 6 groups, 8 rats each: Group-I kept untreated and were administered milk and considered as a control group. Group-II kept as untreated-osteoporosed control group where osteoporosis was induced by I.M. injection of dexamethasone in a dose of 7 mg/kg b.w., as described [18]. Group-III or alendronate treated group. According to the body surface area table [19], the rat dose in mg/kg was calculated from the human dose of 70 mg/kg, by multiplying the human dose by 0.018. Accordingly, the rat dose was equal to 1.26 mg alendronate per 200 g rat. One tablet of alendronate 70 mg (Aesica pharmaceuticals GmbH) was ground and dissolved in 55.5 ml distilled water. Osteoporosed animals were administered alendronate orally by a stomach tube in 0.5 ml of the alendronate stock solution per 100 g b.w. of rat, for 8 weeks. Therefore, the final dose in v/w was 0.5 ml/100 g b.w. for each rat. Group-IV or olive oil treated group where osteoporosed animals received olive oil (Isis Company, Cairo, Egypt) daily in a dose of 1 ml/100 g b.w., orally by a stomach tube for 8 weeks [20]. Group-V or Lepidium sativum treated group where osteoporosed animals received ground Lepidium sativum seeds (Local commercial, Cairo, Egypt) suspended in warm milk which was freshly prepared daily in a dose of 0.12 g/100 g b.w., orally for 8 weeks. This dose was a trial dose in this experimental design. The dose was 12.6 g of ground seeds suspended in full cream commercial milk. Group-VI or “combination treated” group, where osteoporosed animals received a combination of olive oil and *Lepidium sativum* for 8 weeks using the same previously mentioned doses, respectively.

### Sample collection, preparation, and storage

Blood samples were collected from each rat of 8 rats per group at the time of their sacrifice. Blood was received in centrifuge tubes. The samples were then centrifuged at 4000 rpm for 10 min at room temperature. Serum was then separated and stored in Eppendorf tubes and kept at − 20 for biochemical analyses.

### Biochemical analyses

Calcium and inorganic phosphorous were measured using commercial diagnostic kits provided by BioSystems S.A. (Costa Brava, Barcelona, Spain) following the standard protocol. The instrument automatically calculated the concentration of Ca^2+^ in mg/dl (mmol/l). The concentration of phosphorous in blood was expressed as mg/dl (mmol/l). The Rat Osteocalcin/Bone Gla Protein (OT/BGP) ELISA Kit (Sinogeneclon Biotech Co., Ltd., China) was used for estimation of osteocalcin in rat serum following the manufacturer’s instructions. Briefly, osteocalcin was first incubated with the biotinylated antibody in a streptavidin-coated microtiter well then washed and incubated with the HRP conjugated antibody. Following another wash, the enzyme antibody bound to the well was incubated with a substrate solution in a timed reaction and then measured in a spectrophotometric microtiter plate reader. A standard curve was generated and the concentration of osteocalcin in the samples is determined directly from this curve.

### Histopathology and immunohistochemistry

For bone tissue collection, the leg of each animal was disarticulated at the hip, knee, and ankle. Femurs were then removed and immediately placed in 10% formalin. The femur was then cleaned of soft tissue around. Bone (femur) specimens were trimmed and placed in processing cassettes, decalcified for 48 h in a stirred glass beaker containing a solution of 100 ml distilled water and 5 ml concentrated nitric acid, then washed in tap water for 8 h, and transferred to 70% ethanol. Bone specimens were processed and embedded in paraffin and sectioned at 5 µm for conventional histopathological examination. Sections were stained with hematoxylin–eosin (H&E) and visualized by Olympus microscope to study the histopathological changes.

For immunohistochemical staining, tissue sections were deparaffinized in xylene, rehydrated in descending series of alcohol, and incubated in citrate buffer (pH 6.0) solution at 80 °C for 15 min. The sections were then placed in 3% H_2_O_2_ solution for 15 min to block the endogenous peroxidase activity and subsequently incubated at room temperature for 60 min with osteopontin mouse monoclonal antibody (Vector laboratories, CA, USA). The signal was developed by diaminobenzidine to visualize the immunocomplexes. The developed signals were examined using Olympus light microscopy (Olympus, Waltham, MA) and photomicrographs of at least three representative fields were taken by ToupCam digital camera.

### Immunohistochemical evaluations

The immunohistochemically stained slides of bony tissue were examined by using light microscopy. The staining results were scored semiquantitatively according to the Remmele immunoreactive score (IRS). The osteopontin protein expression score was calculated as the product of the percentage of positively stained cells which was divided into five grades of 0–4 (0%, < 10%, 10–50%, 51–80%, and > 80%) and multiplied by the intensity of the immunohistochemical reaction scaled from 0 to 3. Then the obtained IRS was interpreted as 0 to 1 = no expression (negative); 2 to 3 = weak expression; 4 to 8 = moderate expression; and 9 to 12 = strong expression [21].

### Statistical analysis

The data were presented as mean ± standard error of the mean (SEM). The software SPSS version 20.0 (SPSS Inc., Chicago, IL) was used in statistical analyses and significance of the data was considered at *p*-value of less than 0.05. The *t*-test was used to compare dexamethasone-induced versus normal rats and ANOVA for comparison of multiple treated groups.

## Results

### Serum Ca^2+^, P^3+^, and osteocalcin levels in osteoporosed rats treated with alendronate, olive oil, Lepidium sativum, individually and in combination

**Fig. 1:**
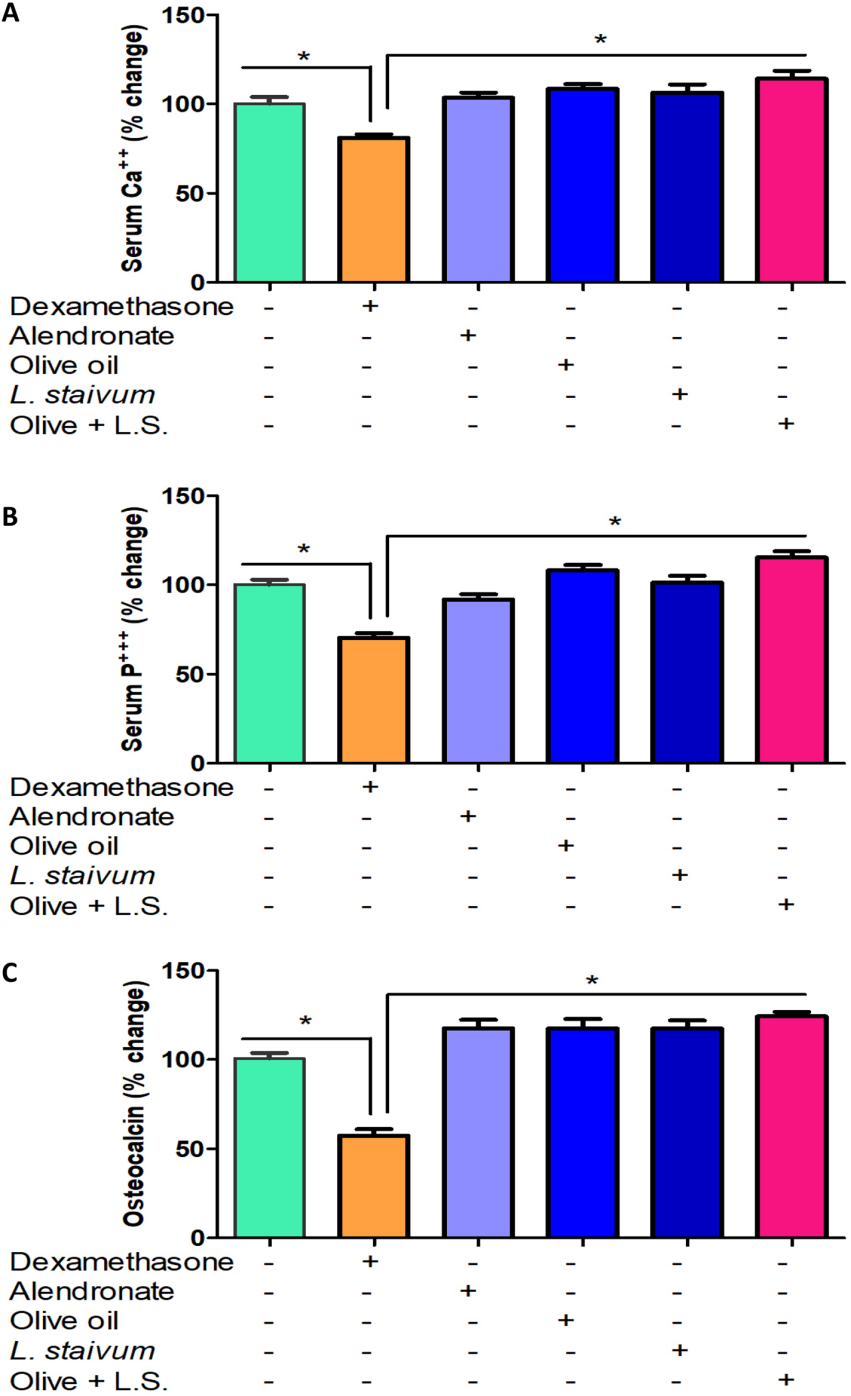
**A**–**C** Effect of olive oil and Lepidium sativum on serum level of Ca^2+^ (**A**), P^3+^ (**B**) and osteocalcin (**C**) in osteoporosed rats. Rats (*n* = 8 per group) were treated with alendronate, olive oil, and Lepidium sativum, in osteoporosis-induced rat model. *Depicts significance at *p* < 0.05 compared to healthy control rats and ^#^regarding osteoporosed animals. LS: Lepidium sativum. Each parameter was measured in 8 rats per each group in triplicates and data represented as mean ± SEM

As illustrated in Fig. 1A and Table 1, the level of serum Ca^2+^ was significantly decreased (*p* < 0.05) in the osteoporosed rats (7.74 ± 0.09) as compared to the control healthy group (9.53 ± 0.21). There was no significant difference in the level of Ca^2+^ of rats treated with alendronate (9.88 ± 0.20) or Lepidium sativum groups (10.14 ± 0.22), as compared to the control group (9.53 ± 0.21). However, there was a significant increase in the level of serum Ca^2+^ in rats treated with olive oil (10.35 ± 0.34) and in combination with Lepidium sativum (10.91 ± 0.31) as compared to control rats. Nevertheless, all treated groups showed a significant increase in serum Ca^2+^ level in alendronate (9.88 ± 0.20), olive oil (10.35 ± 0.34), Lepidium sativum (10.14 ± 0.22), and in combination of olive and Lepidium sativum (10.91 ± 0.31) group as compared to the osteoporosed group (7.74 ± 0.09). The combination of olive oil and Lepidium sativum showed a significant increase in serum Ca^2+^ level as compared to alendronate treated group. Regarding phosphorous level in the serum of rats, there was a significant decrease in its level (*p* < 0.01) in the osteoporosed (3.7 ± 0.18) as compared to the control groups (5.26 ± 0.19). There were no significant differences in serum P^3+^ level in alendronate (4.83 ± 0.15), olive oil (5.69 ± 0.58), and Lepidium sativum (5.33 ± 0.14) treated animals with regard to the control group (5.26 ± 0.19). However, the combination group (6.08 ± 0.09) showed a significant increase in level of phosphorus as compared to control group (5.26 ± 0.19) as shown in Table 1 and Fig. 1B. Table 1 and Fig. 1C show that a significant decrease (*p* < 0.05) in the serum osteocalcin level of the osteoporosed group (1.04 ± 0.05) as compared to the control group (1.81 ± 0.09). When rats treated with alendronate (2.13 ± 0.13), olive oil (2.13 ± 0.13), Lepidium sativum (2.13 ± 0.08), and a combination (2.25 ± 0.13), the level of osteocalcin was significantly increased (*p* < 0.05) as compared to the control group (1.81 ± 0.09).

**Table 1:** Serum Ca^2+^, P^3+^, and osteocalcin levels in osteoporosed rat model treated with olive oil and Lepidium sativum, individually and in combination, using alendronate as authenticated drug for treatment of osteoporosis

Group	Control (*n* = 8)	Osteoporosis (*n* = 8)	Alendronate (*n* = 8)	Olive oil (*n* = 8)	*L. sativum* (*n* = 8)	Olive oil + Lepidium sativum (*n* = 8)
Calcium	9.53 ± 0.21	7.74 ± 0.09*	9.88 ± 0.20^#^	10.35 ± 0.34^#^	10.14 ± 0.22^#^	10.91 ± 0.31^#&^
Phosphorous	5.26 ± 0.19	3.7 ± 0.18*	4.83 ± 0.15^#^	5.69 ± 0.58^#&^	5.33 ± 0.14^#^	6.08 ± 0.09^#&^
Osteocalcin	1.81 ± 0.09	1.04 ± 0.05*	2.13 ± 0.13^#^	2.13 ± 0.13^#^	2.13 ± 0.08^#^	2.25 ± 0.13^#^

Ca^2+^, P^3+^, and osteocalcin were measured in 8 rats per group in triplicates. Data presented as mean ± SEM

Data represented as mean ± SEM

^*^Significant results as compared to control group

^#^Significant results as compared to osteoporosed group

^&^Significant results as compared to alendronate drug group

^*, #, &^Depicts significance at *p* < 0.05

### Effect of treatment of olive oil and Lepidium sativum, individually and in combination on cortical bone thickness in osteoporosed rat model

There was a significant decrease in cortical bone thickness of the osteoporosed group (0.05 ± 0.003) as compared to healthy control group (0.13 ± 0.005), depicted in Table 2. On the contrary, rats treated with alendronate (0.09 ± 0.005), olive oil (0.08 ± 0.006), and Lepidium sativum (0.10 ± 0.008) or in combination (0.17 ± 0.009) showed a significant increase (*p* < 0.05) in cortical bone thickness as compared to the osteoporosed group (0.05 ± 0.003).

**Table 2:** Effect of treatment of olive oil and Lepidium sativum, individually and in combination on cortical bone thickness in osteoporosed rat-model

Group (*n* = 8)	Mean ± SE (mm)
Control	0.13 ± 0.005
Osteoporosis	0.05 ± 0.003^*^
Alendronate	0.09 ± 0.005^#^
Olive oil	0.08 ± 0.006^#^
*L. sativum*	0.10 ± 0.008^#^
Olive oil and Lepidium sativum	0.17 ± 0.009^#&^

Cortical bone thickness was determined in 8 rats per group from at least three independent sections. Data presented as mean ± SEM

^*^Significant results as compared to control group

^#^Significant results as compared to osteoporosed group

^&^Significant results as compared to alendronate drug group

^*, #, &^Depicts significance at *p* < 0.05

### Histopathological and immunohistochemical changes after treatment of osteoporosed rats with olive oil and Lepidium sativum, individually and in combination

**Fig. 2:**
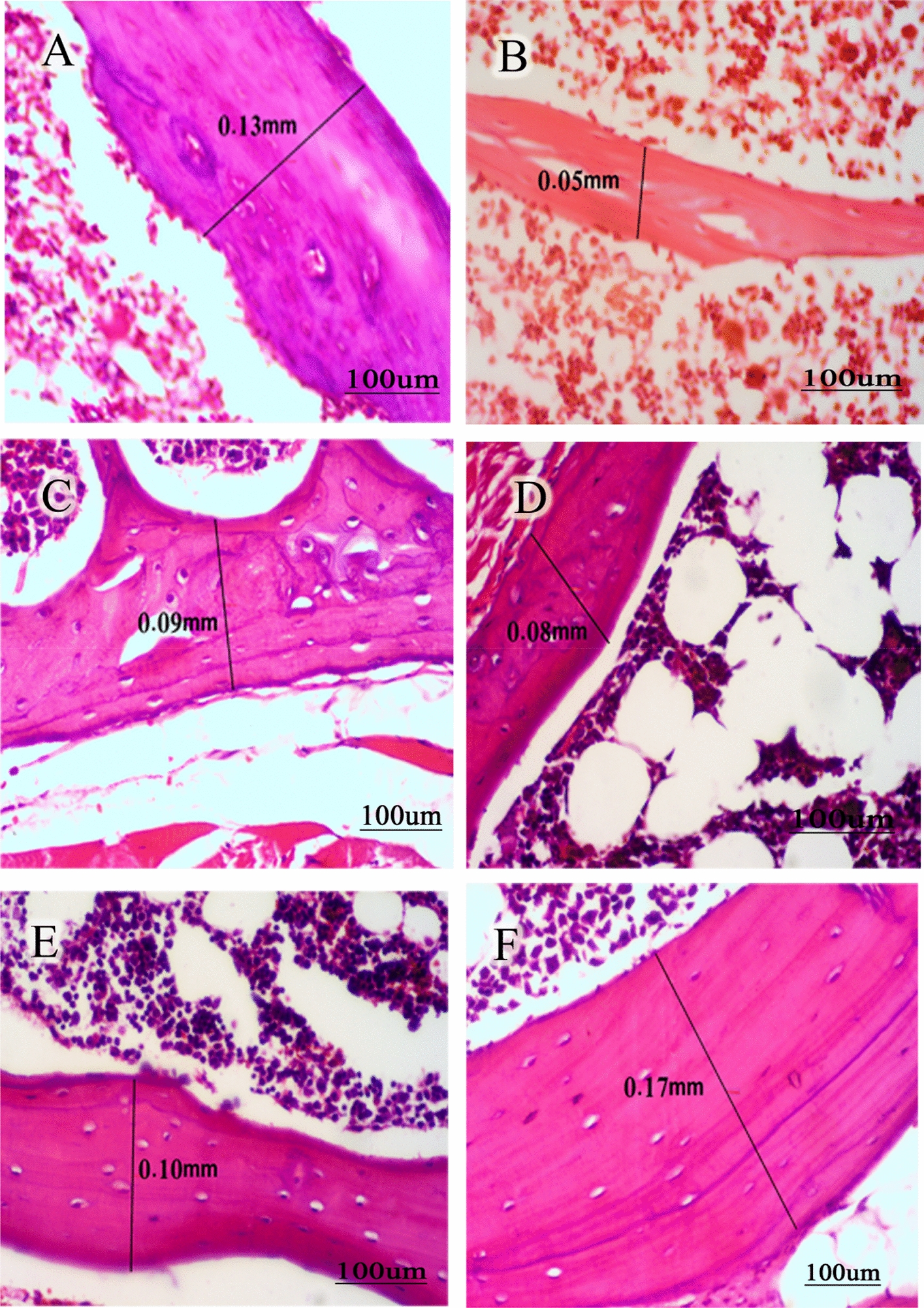
Histopathology of femur in osteoporosis rat model after treatment with a combination of olive oil and Lepidium sativum. Representative bone sections collected from 8 rats were stained with hematoxylin and eosin. **A** Control group presented with cortical bone thickness of 0.13 mm. **B** The osteoporosed group has a cortical bone thickness of 0.05 mm. **C** The alendronate treated group presented with cortical bone thickness of 0.09 mm. **D** The olive oil treated group presented with cortical bone thickness of 0.08 mm. **E** The Lepidium sativum group presented with cortical bone thickness of 0.10 mm. **F** The combination group presented with cortical bone thickness of 0.17 mm. Magnification was 400×

Cortical bone thickness in bony specimens was histopathologically examined. Histological examination showed that there was a significant decrease in the cortical bone thickness in osteoporosed rat model as compared to healthy group (Fig. 2A and B). Treatment of rats with Lepidium sativum reversed the dexamethasone effect in the osteoporosed group better than the effect of olive oil and alendronate (Fig. 2C–E). The combination of olive oil and Lepidium sativum treated group showed a marked increase in the cortical bone thickness as compared to all other individually treated groups (Fig. 2F).

**Fig. 3:**
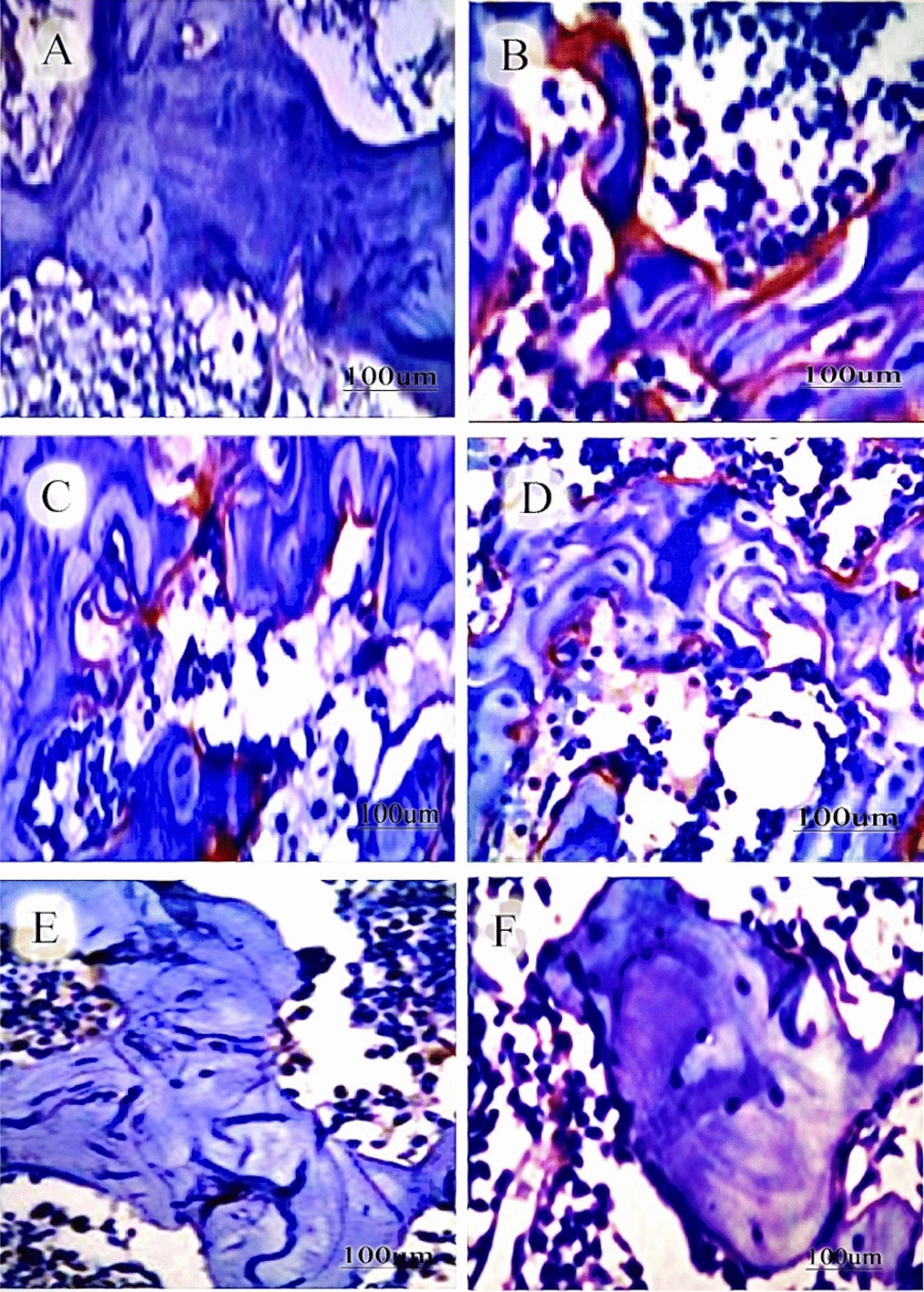
Immunohistochemical studies of osteopontin expression in bone tissues collected from osteoporosis-induced rats treated with a combination of olive oil and Lepidium sativum. **A** A representative micrograph of control group (8 rats) shows a negative expression for osteopontin. **B** The osteoporosed group shows a moderate positive expression of osteopontin. **C** The alendronate treated group indicates a moderate positive expression of osteopontin. **D** The olive oil treated group with a low positive expression of osteopontin. **E** The Lepidium sativum treated group with a moderate positive expression of osteopontin. **F** The combination group with a weak expression of osteopontin. Scale bar was 100 µm

Immunostaining of bone tissues showed a remarkable induction of osteoporosis in rats. This was reflected by a significant increase (*p* < 0.05) in osteopontin protein expression in bone tissues. Treatment of rats with a combination of olive oil and Lepidium sativum exhibited a significant decrease in osteopontin staining (*p* < 0.05) compared to individual treatment and osteoporosed animals as elucidated in Fig. 3.

## Discussion

Osteoporosis is a metabolic disease of bone characterized by the mechanical impairment of the bone tissue and loss of bone mass associated with an increase in weakness of the bone and tendency to fracture [22, 23]. Synthetic glucocorticoids are used for treatment of many immune and inflammatory disorders. It was reported that the most common side effect of prolonged use of glucocorticoid therapy is secondary osteoporosis [24]. One of the proposed mechanisms for bone resorption by glucocorticoids includes activation of important kinase systems [25, 26], reduced production of osteoprotegerin, and increased production of receptor activator of nuclear factor-kB ligand (RANKL), leading to increase of osteoclast recruitment and survival [27, 28]. As essential minerals, Ca^2+^ and P^3+^ participate in development of skeletal system in our body, maintain bone density, prevent bone loss, and lower the risk of fracture. To predict any bone abnormalities, serum osteocalcin is a golden biochemical marker for bone formation. In this study, the combination effect of virgin olive oil and Lepidium sativum was evaluated in an osteoporosis model of dexamethasone-induced rats. Our results showed that treatment of osteoporosed rats with a combination of olive oil and Lepidium sativum significantly increased the level of serum Ca^2+^, P^3+^, and osteocalcin compared to individual treatment of rats. Alendronate sodium was used as an authenticated control, which is FDA-approved drug for treatment of osteoporotic patients [29].

One study reported the same findings in rats treated with olive oil in which serum calcium level was increased [30]. Virgin olive oil contains a wide variety of active compounds, which have a protective and therapeutic effect on osteoporosis [31]. These components are suggested to benefit bone formation by acting on bone cell metabolism [32]. Other studies showed that olive oil is an excellent source of gamma-linolenic acid (GLA), which is known to reduce the excretion of calcium, inhibits bone resorption, and decreases markers of bone turnover [8, 33]. Oleuropein, the main phenolic compound of olive oil, enhances bone health via stimulating osteoblasts formation by bone marrow stem cells and decreases adipocytes generation. In addition, the same authors reported that oleuropein intake has a preventive effect against osteoporosis and aging-related bone loss. Olive oil contains caffeic acid, p-coumaric acid and ferulic acid, which increase the level of estradiol in blood [34]. Hydroxytyrosol has been identified as one of the most potent antioxidants within olive oil [35]. Tyrosol and hydroxytyrosol administration prevented the inflammation-induced bone mass loss in a senile ovariectomized osteoporotic rat model [36]. It was reported that luteolin decreases the differentiation of bone marrow mononuclear cells into osteoclasts and inhibits the bone resorptive activity of differentiated osteoclasts [14, 37]. Another study, conducted in 2007, demonstrated that luteolin increases collagen synthesis, osteocalcin secretion, and alkaline phosphatase (ALP) activity as indicated in the current study.

Concerning the Lepidium sativum treated animals, the level of serum Ca^2+^ and P^3+^ was increased as compared to the osteoporosed group. Other independent studies reported an improvement in serum Ca^2+^ level [38]. Coetzee et al. have shown that Lepidium sativum has a high content of omega-3 fatty acids, which in turn increases Ca^2+^ absorption in the small intestine from the enterocytes. These enterocytes are exposed to eicosapentaenoic acid, docosahexaenoic acid, or arachidonic acid. Interestingly, docosahexaenoic acid is the only agent which was able to increase the basal Ca^2+^ absorption [39]. The next expected step is more Ca^2+^ will be incorporated into mineral matrix of the bone. Moreover, docosahexaenoic acid upregulates osteoblast maturation, leading to an increase in osteoblast population, and resulted in stronger bones [16, 40]. Arachidonic acid increases soluble and membrane bound to RANKL production by osteoblasts [39]. Subsequently, this stimulates osteoclast differentiation followed by an increase in bone resorption. On the contrary, docosahexaenoic acid inhibits osteoclast maturation, and may indirectly prevent arachidonic acid stimulated RANKL production [41]. Other studies also highlighted the potential role of polyunsaturated fatty acid in modulating the inflammatory reactions of bone remodeling [42, 43]. *Lepidium sativum* has beneficial effects on increasing bone density and mineralization. As indicated by biochemical results of this study, the increased bone density might be due to the seed’s rich content of calcium and essential fatty acids that improve general bone health. In parallel, the combination of olive oil and Lepidium sativum provides a better effect on bone remodeling. This was reflected by their effects on the inflammatory processes, through the increase of osteoblastic and/or decrease of the osteoclastic activity. This was evidenced by a decrease in the cortical thickness of the femur in the osteoporosed-induced group compared to the control group [44, 45]. In the current study, the bone sections of the osteoporosed rats revealed outer cortical bone thinning with the presence of fissures. In addition, there was a deteriorated architecture of the trabecular bone. However, the group treated by Lepidium sativum revealed a marked improvement in the cortical bone thickness, as compared to the osteoporosed group, in accordance with a prior study [38]. Interestingly, there was a significant increase in the cortical bone thickness in rats treated with a combination of Lepidium sativum and olive oil as compared to alendronate treated animals. Collectively, these results indicate that the combination of the two natural products suppressed more efficiently the bone resorption than the individual treatments and improved bone mineral health and intensity in a rat model of osteoporosis.

## Data Availability

All data are available.
